# Surface-Engineered Magnetic Nanoparticles in Skeletal Muscle Tissue Engineering: From Biological Interactions to Clinical Translation

**DOI:** 10.3390/mi17080912

**Published:** 2026-07-29

**Authors:** Md Imran Hossain, Sitansu Sekhar Nanda, Dong Kee Yi

**Affiliations:** 1 Deakin Institute for Frontier Materials, Faculty of Science, Engineering and Built Environment, Deakin University, Geelong, VIC 3216, Australia; mdimran.h@research.deakin.edu.au; 2 Department of Chemistry, Myongji University, Yongin 17058, Republic of Korea; nandasitansusekhar@gmail.com

**Keywords:** tissue engineering, magnetic nanoparticles, magnetic drug delivery, magnetic imaging, skeletal muscle regeneration, magnetic hyperthermia

## Abstract

The repair and functional restoration of skeletal muscle tissue following trauma, degenerative disease, or volumetric muscle loss remains a significant unmet clinical challenge in tissue engineering, where the need to recapitulate the anisotropic architecture, mechanical compliance, and high metabolic demands of native muscle imposes stringent requirements on biomaterial design. Traditional cell culturing and scaffold fabrication strategies have proven insufficient to address these demands in isolation, particularly in integrating mechanical integrity, biochemical functionality, and biological activity within a single biomaterial system. Recent advances in material science have accelerated the evolution of skeletal muscle tissue engineering toward a more precise and technologically sophisticated discipline. In this context, surface-engineered magnetic nanoparticle (MNP) hybrids have emerged as a promising multifunctional platform, owing to their intrinsic biocompatibility, tunable physicochemical properties, and rapid, non-invasive responsiveness to external magnetic fields. These unique characteristics have enabled the development of magnetic force-based tissue engineering strategies, facilitating controlled myogenic cell organization, magnetically guided delivery of therapeutic agents and stem cells, enhanced muscle construct formation within responsive scaffolds, and real-time non-invasive monitoring of engineered systems via MRI. This review systematically synthesizes the recent advances in surface-engineered MNP platforms for skeletal muscle tissue engineering, covering organic and inorganic coating strategies, magnetically responsive scaffold integration, guided cell and drug delivery, and construct monitoring, whilst critically appraising the biocompatibility, biodistribution, and regulatory challenges that currently define the translational pathway for MNP-augmented skeletal muscle constructs.

## 1. Introduction

**Figure 1:**
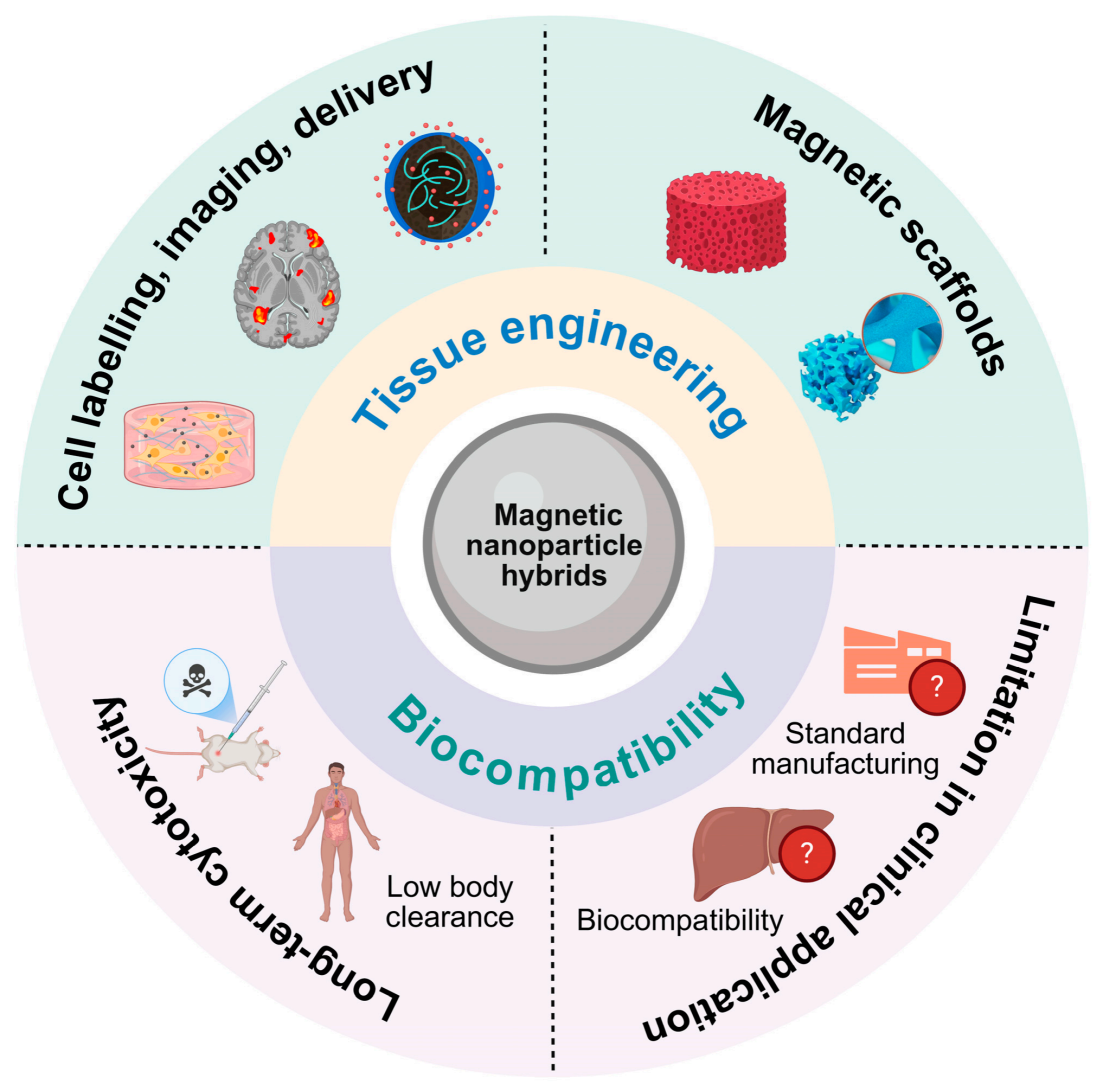
Overview and current limitations of magnetic nanoparticle hybrids in tissue engineering and biomedical therapeutics.

Skeletal muscle tissue engineering demands biomaterial platforms capable of simultaneously providing structural guidance, biochemical signaling, and real-time monitoring requirements that conventional scaffolds have struggled to fulfill. This review examines how surface-engineered magnetic nanoparticles address these demands, from coating design and scaffold integration through to clinical translation. Replicating the complexity of native tissues in vitro remains a major tissue engineering (TE) challenge [1]. Traditional cell culturing and harvesting methods rely on harsh enzymatic or chemical agents, such as trypsin and EDTA, which disrupt crucial interactions between cells and the extracellular matrix (ECM) necessary for healthy tissue development [2]. To address this, the introduction of stimuli-responsive materials has enabled safe, damage-free cell detachment without relying on destructive proteases [3]. Building on these advances, magnetic-responsive systems have become powerful tools in regenerative medicine; specifically, magnetically labeled cells allow researchers to fabricate tissue replacements with exact control over cellular positioning [4]. By applying an external magnetic field, these nanoparticle-labeled cells can be precisely guided to specific locations to stimulate tissue repair and restore biological function [5]. Additionally, the inherent magnetic properties of these bioengineered materials enable continuous, non-invasive tracking over time, a concept demonstrated in numerous preclinical MRI studies aimed at regenerating damaged tissues [6]. Although contemporary human clinical trials have confined the application of magnetic fields to monitoring, this review synthesizes major advances from the past 5 years in the use of magnetic-responsive systems for tissue engineering, specifically for noninvasive construct tracking, magnetically guided targeted cell delivery, and magnetically actuated approaches to active tissue regeneration. Figure 1 schematically summarizes the principal recent applications of magnetic nanoparticles in tissue engineering and biomedical therapeutics discussed in this review.

## 2. Surface Engineering of MNPs

Magnetic nanoparticles (MNPs) represent a structurally and compositionally diverse class of nanomaterials unified by their responsiveness to external magnetic fields. For biomedical and tissue engineering applications, MNPs are broadly classified by chemical composition, magnetic behavior, and structural architecture.

Iron oxide nanoparticles, principally magnetite (Fe_3_O_4_) and maghemite (γ-Fe_2_O_3_), constitute the most extensively studied and clinically translated subclass of MNPs [7]. Their regulatory approval history, well-characterized biodegradation pathways via endogenous iron metabolism, and tunable magnetic properties make them the dominant platform for biomedical development [8]. Spinel ferrites of the general formula MFe_2_O_4_ (where M = Mn, Co, Ni, Zn, Cu) offer compositionally tunable magnetic properties. Manganese ferrite (MnFe_2_O_4_) exhibits high MRI T_1_/T_2_ contrast sensitivity [9], while cobalt ferrite (CoFe_2_O_4_) demonstrates high magnetocrystalline anisotropy suitable for magnetic hyperthermia [10]. Zinc-substituted ferrites have been explored for enhanced saturation magnetization [11]. Pure metallic MNPs, including iron (Fe^0^), cobalt (Co), and nickel (Ni), possess higher saturation magnetization values than their oxide counterparts but are clinically limited by oxidative instability and cytotoxicity [12]. FeCo and FePt alloy nanoparticles have attracted interest for high-performance magnetic applications, though biological translation remains constrained [13]. Gadolinium-based nanoparticles and rare-earth-doped iron oxides have been explored for multimodal imaging, combining MRI contrast enhancement with optical or photoacoustic modalities [14].

A wide range of surface-engineering strategies has been developed as well, including polymer, lipid, and biomimetic coatings, as well as silica, gold, and carbon-based shells, each offering distinct advantages for stabilizing and functionalizing magnetic nanoparticles. Poly(ethylene glycol) (PEG) functionalization, PEGylation, remains the most established strategy for conferring colloidal stability and immune evasion to MNPs [15]. The dense PEG brush layer sterically inhibits opsonin adsorption and reticuloendothelial system (RES) uptake, substantially extending systemic circulation time [16]. Recent work has explored brush density optimization, PEG molecular weight effects, and zwitterionic alternatives to address PEG immunogenicity concerns [17]. Poly(lactic-co-glycolic acid) (PLGA) encapsulation of MNPs enables simultaneous drug encapsulation and sustained release in a biodegradable matrix [18]. Chitosan, a naturally derived polysaccharide, has been extensively explored for MNP coating due to its mucoadhesive properties, inherent antibacterial activity, and amine-rich surface amenable to further conjugation [19].

While inorganic shells such as silica and gold provide chemical stability and photothermal functionality, organic coatings represent the most clinically established and compositionally diverse category of MNP surface modifications, serving the dual function of improving biocompatibility and critically modulating the intrinsic magnetic properties of nanoparticle cores that ultimately govern diagnostic and therapeutic performance. At the nanoparticle surface, translational symmetry breaking generates coordinatively unsaturated atoms whose disordered spin states degrade saturation magnetization, MRI contrast efficiency, and hyperthermia output. Organic ligands actively remediate this disorder by binding to surface metal atoms, replacing missing coordination bonds, and restoring the bulk crystal environment, simultaneously reducing surface spin canting and recovering saturation magnetization [20]. The denticity and surface coverage of organic molecules critically determine these effects: in EDTA-functionalized γ-Fe_2_O_3_ nanoparticles, chelating EDTA groups directly controlled the spin-glass-like surface layer thickness and canted Fe spin fraction, with larger organic surface fractions suppressing exchange bias, whilst smaller fractions retained magnetically disordered surface layers [21]. At the ensemble level, organic coatings regulate interparticle dipolar coupling by physically separating nanoparticle cores. Polysaccharide-coated ferrihydrite nanoparticles demonstrated substantially reduced interparticle interaction energies and modified blocking temperatures compared to uncoated counterparts, confirming that coating architecture governs ensemble-level magnetic behavior [22].

Additionally, liposomal and lipid bilayer encapsulation yield magnetoliposomes that combine the biocompatibility of lipid membranes with the magnetic responsiveness of the core [23]. Cell membrane camouflage coating MNPs with erythrocyte, platelet, macrophage, or cancer cell-derived membranes represents an emerging biomimetic strategy to evade immune clearance and exploit cell-specific targeting mechanisms [24]. Silica (SiO_2_) shells are among the most widely employed inorganic coatings, providing chemical stability, ease of surface functionalization via well-established silane chemistry, and optical transparency [25]. Mesoporous silica-coated MNPs (e.g., Fe_3_O_4_@mSiO_2_) offer high drug loading capacity through their ordered pore structure and are amenable to stimuli-responsive gating mechanisms [26]. Gold-shell functionalization (Fe_3_O_4_@Au) confers photothermal conversion capability, enabling combined magneto-photothermal therapy, while simultaneously providing a thiol-reactive surface for facile bioconjugation of antibodies, peptides, and nucleic acids [27]. Beyond passive coatings, the surface engineering toolkit encompasses covalent and non-covalent conjugation of bioactive molecules, including targeting ligands, therapeutic payloads, imaging agents, and enzyme or protein functionalization strategies, each conferring distinct biological or diagnostic functionality to the MNP platform. Depending on their intended application, surface-engineered MNPs can be broadly categorized into two functional domains. The first encompasses platforms designed for tissue engineering and regenerative medicine, where surface modifications are principally directed toward scaffold integration, magnetically guided cell delivery, and tissue-specific regenerative outcomes; representative examples are summarized in Table 1. The second encompasses theranostic and imaging platforms, where surface engineering prioritizes tumor targeting, multimodal contrast enhancement, and stimuli-responsive drug release.

The surface engineering strategies outlined above, spanning polymer coatings, biomimetic membranes, inorganic shells, and bioactive molecule conjugation, are not merely physicochemical refinements aimed at improving colloidal stability or immune evasion in systemic circulation. Rather, they constitute a foundational prerequisite for the successful integration of MNPs into functional tissue-engineering scaffolds. The biocompatibility, targeting specificity, and stimulus responsiveness conferred by these surface modifications directly govern MNPs’ performance when embedded in hydrogel matrices, electrospun fibrous scaffolds, and 3D-printed constructs. Specifically, hydrophilic coatings such as PEG and dextran maintain colloidal dispersion within scaffold pore networks; biodegradable polymer shells such as PLGA enable controlled therapeutic release within the scaffold microenvironment; and biomimetic cell-membrane coatings reduce inflammatory activation at the implantation site, all of which are critical determinants of scaffold-level biological performance, as discussed in the sections that follow.

## 3. Magnetic Nanoparticle-Based Tissue Engineering

TE aims to restore the structure and function of damaged tissues or organs arising from trauma, degenerative processes, or disease [34]. Achieving constructs that faithfully reproduce the intricate architecture of native tissues is essential for ensuring their physiological performance. Conventional TE strategies predominantly rely on a top-down paradigm, in which cells are introduced onto a pre-fabricated scaffold designed to be biocompatible and biodegradable [35]. Once seeded, cells infiltrate the scaffold, deposit their own extracellular matrix (ECM), and gradually establish an appropriate microenvironment. Mechanical cues, perfusion systems, and the incorporation of bioactive molecules, including growth factors and signaling agents, are commonly employed to support tissue maturation within these constructs [36]. A variety of fabrication techniques, such as freeze-drying [37], solvent casting [38], electrospinning [39], and supercritical fluid processing [40], have been widely used to generate top-down scaffolds. This approach has enabled the successful engineering of relatively thin or avascular tissues, including skin, cartilage, and bladder [41]. However, recent advances in magnetic nanoparticle–based tissue engineering have generated significant interest in the field. Current strategies leverage magnetically responsive systems for cell labeling and imaging, magnetically guided drug or factor delivery, and scaffold-integrated magnetic platforms, all of which represent major avenues for enhancing tissue regeneration.

### 3.1. Magnetically Responsive Systems for Cell Labeling, Imaging, and Controlled Delivery

In regenerative medicine, replacement tissues may be fabricated in advance and implanted, or they may be assembled directly at the injury site. In either case, the implanted constructs must be continuously monitored to assess their maturation and functional performance. Consequently, the development of reliable and noninvasive monitoring methodologies is essential for the successful clinical translation of engineered tissues. Effective regeneration requires detailed evaluation of the evolving tissue’s structure, composition, and mechanical integrity throughout the healing process [42].

Magnetic-force-assisted cell-patterning platforms have been developed to integrate magnetic manipulation with micropatterning technologies for tissue engineering applications. Owing to their high spatial precision, these systems enable the creation of single-cell arrays that facilitate the investigation of individual cell behavior, morphological dynamics, and cell–cell communication, with potential relevance for diagnostic studies and mechanistic analyses. Among the most widely explored configurations are devices containing regularly spaced micropillars, which serve as anchoring points for magnetically responsive cells. For instance, Ino et al. designed a magnetic holder incorporating an array of pillars that attracted magnetically labeled cells to defined positions [43]. This platform was subsequently used to examine cellular organization and function during angiogenic processes. By guiding magnetized cells into predetermined patterns, the system promoted the formation of localized cell clusters and branched networks, highlighting the importance of controlled cell–cell interactions in angiogenesis.

**Figure 2:**
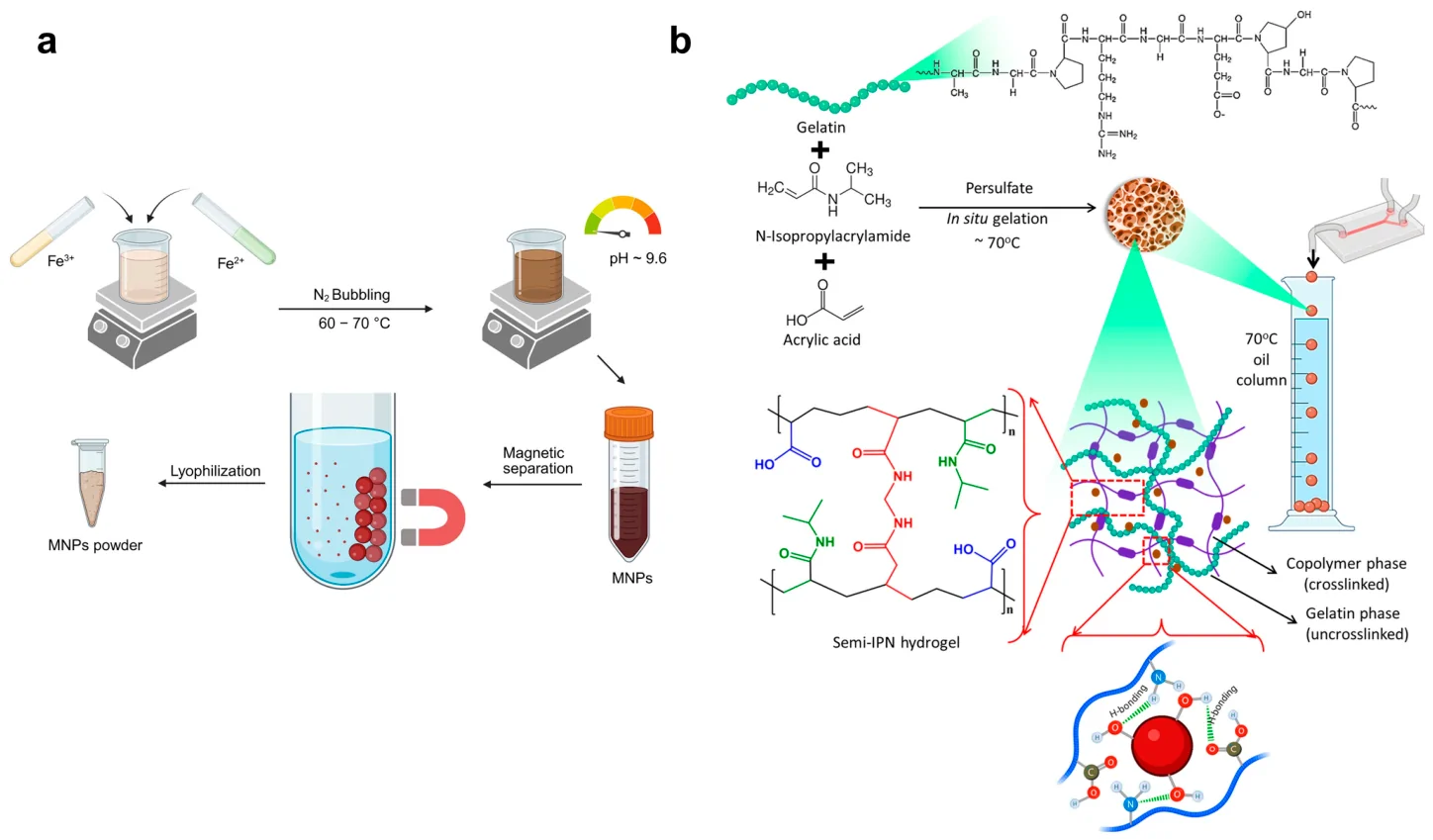
Comprehensive schematic of the synthesis and fabrication pipeline. (**a**) The chemical coprecipitation method was used to prepare iron oxide (Fe_3_O_4_) nanoparticles (redrawn). (**b**) Detailed diagram of the pressure-driven, water-in-oil microfluidic setup employed for the in situ gelation and structuring of the magneto-responsive hydrogel microbeads. Reprinted with permission of the ref. [30].

Additionally, recent innovations in microfluidic-assisted fabrication have enabled the development of highly tunable, stimuli-responsive biomaterials for advanced biomedical applications (Figure 2a). For instance, a recent study reported the synthesis of magneto-responsive, semi-interpenetrating polymer network (semi-IPN) hydrogel microbeads by integrating iron oxide (Fe_3_O_4_) nanoparticles into a gelatin and vinylic monomer matrix. By using a pressure-mediated droplet method coupled with in situ gelation in a heated oil column, the researchers achieved precise control over gelation kinetics and the resulting pore-size distribution (Figure 2b). These nanocomposite microbeads demonstrated robust superparamagnetic properties, superior mechanical elasticity compared to unfilled hydrogels, and reversible, magnetically tunable swelling behaviors. Crucially, the beads’ distinct porous and wrinkled surface morphology facilitated excellent in vitro cell adhesion and proliferation, highlighting their significant potential as a multifunctional platform for magnetically guided therapies, cell delivery, and responsive tissue engineering [30]. Conventional monitoring techniques, such as optical and fluorescence microscopy, provide useful information but are limited by shallow imaging depth. Even advanced modalities, such as multiphoton microscopy, suffer from limited penetration. Although microcomputed tomography can overcome depth limitations, it relies on ionizing radiation and requires dense tissues to generate sufficient contrast [44]. Repeated exposure to ionizing radiation, even at low doses, poses health risks through mechanisms involving reactive oxygen species generation, altered gene expression, and both genetic and epigenetic DNA damage [45].

MRI has already been applied to a wide range of engineered tissues, including cardiac, neural, renal, osseous, cartilaginous, pancreatic, and musculoskeletal constructs. Parameters commonly assessed include oxygen and nutrient diffusion, cellular metabolism, and ECM deposition. Additional tissue-specific features may also be required; for instance, cartilage constructs must demonstrate appropriate mechanical strength, viscoelasticity, and synthesis of collagen II and proteoglycans, whereas bone constructs require matrix deposition, mineralization, angiogenesis, and high load-bearing capacity. Lee et al. comprehensively reviewed the precise synthetic methodologies used to fabricate iron oxide-based magnetic nanoparticles optimized for MRI applications [46].

**Figure 3:**
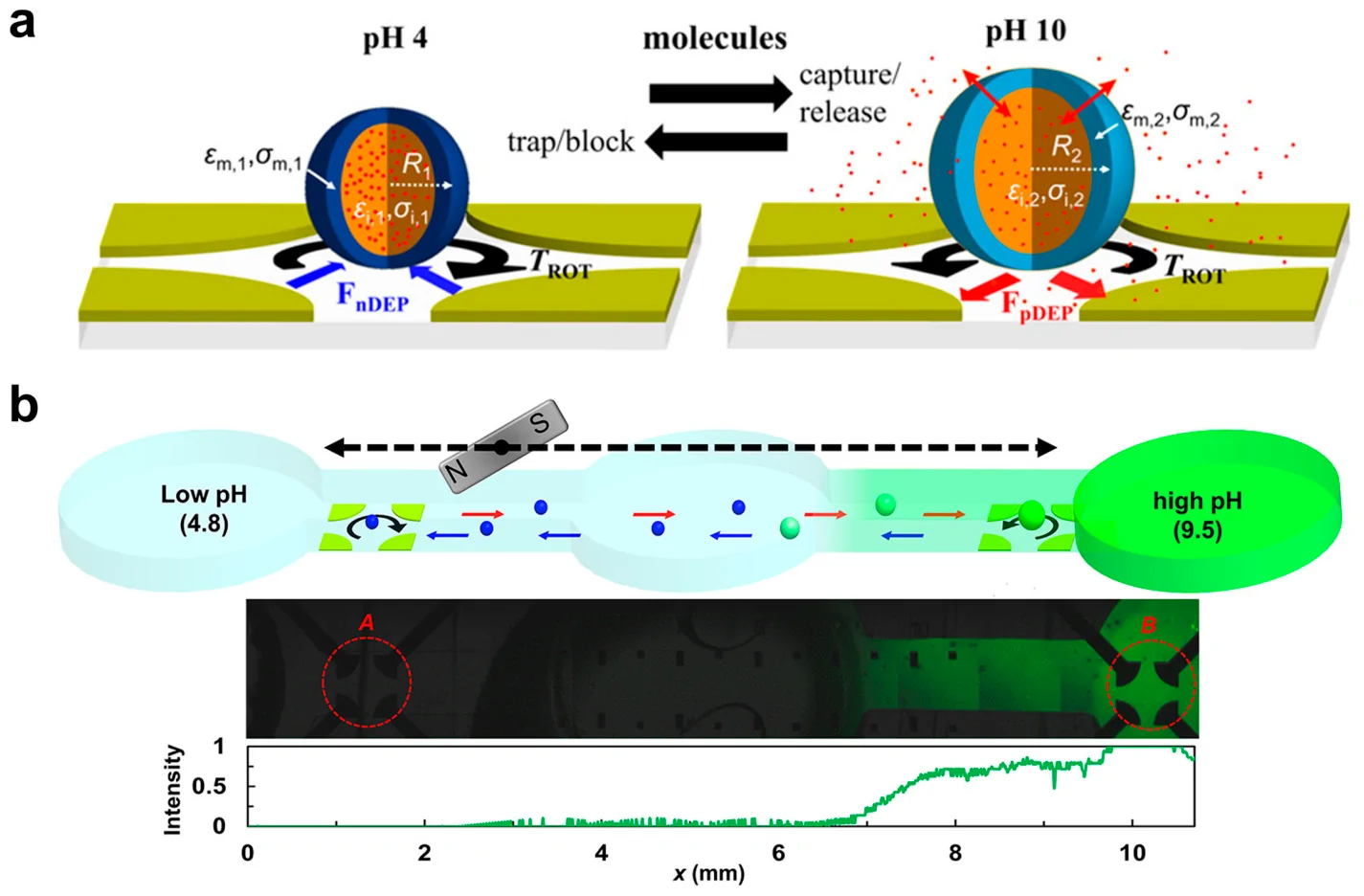
(**a**) Controlled release mechanism under different pH conditions. (**b**) Characterization of magnetic microcapsule dynamics during pH-dependent transport. (**Top**) Schematic representation of microcapsule alterations and ROT direction at 1 MHz across varying pH environments. (**Middle**) Superimposed bright-field and fluorescence micrograph of the fluidic channel. (**Bottom**) Normalized fluorescence intensity profile, with baselines of 0 and 1 corresponding to pH 4.8 (without FITC-Dextran) and pH 9.5 (with FITC-Dextran) solutions, respectively. Reprinted with permission of the ref. [47].

However, to enhance contrast efficiency and targeted therapeutic outcomes, contemporary research has increasingly pivoted toward advanced functionalized magnetic architectures, such as magnetic graphitic nanocapsules (MGNCs) [47], and red blood cell-encapsulated magnetic nanoparticles [48]. The development of smart, stimuli-responsive architectures has significantly advanced precise cargo delivery by coupling mass transport with sensing. For instance, recent progress has demonstrated magnetic microcapsules capable of a highly controlled release mechanism dictated by ambient pH variations (Figure 3a).

Beyond passive release, the dynamic transport behavior of these microcapsules can be actively manipulated and monitored within fluidic networks; under an alternating electric field at 1 MHz, the microcapsules undergo distinct structural alterations and directional electrorotation (ROT) as they transition between different chemical zones. This pH-dependent transport dynamics was characterized in a microfluidic channel using superimposed bright-field and fluorescence microscopy, in which the real-time release kinetics were quantified from normalized fluorescence intensity profiles of encapsulated FITC-Dextran across a pH gradient spanning 4.8–9.5 (Figure 3b) [47]. Additionally, to address the clinical limitations of rapid clearance and poor biocompatibility inherent to conventional imaging agents, a promising biomimetic strategy encapsulates superparamagnetic iron oxide nanoparticles within red blood cells (RBCs) via hypotonic dialysis. This cellular “Trojan horse” platform, prepared by encapsulation of nanoparticles into red blood cells, exploits the natural deformability and extended circulation of RBCs to provide highly robust, long-lasting cerebral blood volume (CBV)-weighted fMRI signals, yielding a greater than 5-fold increase in signal magnitude over traditional blood oxygenation level-dependent (BOLD) contrast. Notably, the bioengineered nanocarriers maintain stable T2* contrast for more than 30 min at only one-quarter of the standard free-nanoparticle iron dose, while drastically enhancing functional mapping resolution to achieve laminar specificity localized to cortical layer IV. By seamlessly merging the biocompatibility of blood-based vectors with strong magnetic responsiveness, this scalable platform offers a transformative alternative for high-resolution functional neuroimaging, longitudinal neurovascular diagnostics, and targeted theragnostic applications [48].

A major recent development in MNP technology is the emergence of multifunctional nanoplatforms that integrate multiple therapeutic and diagnostic functions within a single construct [49]. These next-generation MNP systems are being engineered to simultaneously support imaging, targeted drug delivery [50], and tissue-engineering functions, thereby enabling more efficient and highly localized therapeutic interventions [51,52]. Another promising direction is the design of biomimetic hybrid constructs that incorporate MNPs into advanced polymers or hydrogel matrices. Such composite materials enhance the mechanical, electrical, and structural properties of scaffolds used in regenerative therapies, particularly in neural and skeletal muscle tissue engineering. The integration of MNPs within biocompatible hydrogels also improves cell adhesion, spreading, and overall cellular performance, ultimately contributing to improved therapeutic outcomes [53].

Additionally, magnetic field-based technologies continue to evolve, enabling increasingly precise and controlled applications within tissue engineering. Recent developments include wearable and implantable devices that generate localized magnetic fields to selectively activate MNPs within the body. These systems offer the potential for real-time therapeutic modulation without the need for invasive procedures [28,29]. Another important advancement is the emergence of real-time monitoring and control platforms that use spatially defined magnetic fields to regulate tissue regeneration. By dynamically adjusting the magnetic environment, these systems can influence key biological processes such as cell proliferation, differentiation, and tissue repair. Such adaptive magnetic control strategies represent a step toward more efficient, responsive, and personalized regenerative therapies [54]. Integrating MNPs with emerging biomedical technologies is anticipated to open new avenues in regenerative medicine. One promising direction is the incorporation of MNPs into 3D bioprinting strategies for muscle tissue engineering. When embedded within bio-inks, MNPs can respond to externally applied magnetic fields, enabling the fabrication of spatially organized scaffolds and guiding cell alignment and tissue morphogenesis with high precision [55]. These platforms span a broad functional landscape from tumor-targeted drug delivery and stimuli-responsive release to multimodal MRI, photothermal, and fluorescence imaging. Representative examples of surface-engineered MNP systems developed for theranostic and imaging applications are summarized in Table 2.

### 3.2. Magnetic Scaffold-Based Tissue Engineering

Conventional scaffold-based strategies in tissue engineering rely on “template” structures that support cell adhesion, proliferation, and the formation of three-dimensional tissues [63]. Over recent decades, advances in material design have significantly expanded the applicability of scaffolds in TE. An ideal scaffold must provide a diverse set of chemical, biochemical, and biophysical cues capable of directing highly specific cellular and tissue-level processes [64]. In addition to shielding cells from external stressors, 3D scaffolds are expected to recreate a biomimetic microenvironment and deliver essential growth and differentiation factors that promote tissue formation [64].

To guide cell adhesion, migration, and proliferation, scaffolds must emulate the ECM of native tissues [65]. ECM-derived molecules such as fibronectin, laminin, and integrin-binding peptides have been incorporated into scaffold surfaces to create synthetic ECM analogues that enhance cell–material interactions [66]. Despite these advances, scaffold-based TE still faces challenges. In porous 3D scaffolds, cells often distribute unevenly, particularly in large constructs, resulting in low initial cell densities [67]. To address this, hydrogels have been engineered to encapsulate cells directly, enabling homogeneous distribution and higher cell densities [68].

Hydrogel microcapsules represent one of the most widely explored microscale encapsulation systems. Their semipermeable membranes allow nutrient and metabolite exchange while shielding nonautologous cells from immune rejection [69]. By assembling these microcapsules into larger constructs, researchers have created clinically relevant, modular TE systems [70]. Given the complex architecture of native tissues, nano- and microstructured materials have been incorporated into hydrogel networks to mimic their heterogeneous organization [71]. Magnetic nanoparticles (MNPs), in particular, have gained attention due to their remote actuation capabilities. By manipulating MNP distribution within hydrogels, researchers can engineer anisotropic, magnetically responsive scaffolds with programmable architectures. These hydrogels have been applied in drug delivery, bioseparation, biosensing, and TE. Magnetic or magneto-mechanical stimulation can modulate cell growth, migration, proliferation, and differentiation, enabling the fabrication of cell-laden constructs with highly ordered features that closely resemble native tissue structures [72]. Magnetic hydrogels have shown promise in regenerating neural, cardiac, skin, cartilage, and bone tissues [73].

For example, incorporating Fe_3_O_4_ nanoparticles and curcumin into N-isopropylacrylamide–methacrylic acid hydrogels reduced doxorubicin-induced cardiotoxicity, demonstrating cardioprotective potential [31]. Muscle regeneration has also been enhanced using ferrogel–alginate scaffolds subjected to periodic magnetic stimulation (5 min at 1 Hz every 12 h), which mechanically activated the biomaterial and promoted tissue repair [32]. Injectable magnetic hydrogels have emerged as an effective platform for delivering stem cells and growth factors directly to injured muscle tissue, with their localization and retention precisely controlled by externally applied magnetic fields. In parallel, electrospun magnetic fibers have been developed to replicate the anisotropic architecture of native muscle ECM, providing structural cues that guide cell alignment and promote myogenic differentiation [74]. Improvements in the mechanical performance of muscle tissue scaffolds have also been achieved through the use of biodegradable polymer composites, which provide structural reinforcement while gradually degrading and integrating into the host tissue [5].

Preclinical studies in widely used rodent models, with comparable muscle physiology and well-established injury systems, have demonstrated the therapeutic potential of MNP-based strategies for muscle repair. MNP-loaded scaffolds implanted into muscle defects have accelerated muscle fiber regeneration, accompanied by upregulated myogenic gene expression. When combined with angiogenic cues such as VEGF, MNPs have also enhanced neovascularization, a key requirement for effective muscle healing [75].

**Figure 4:**
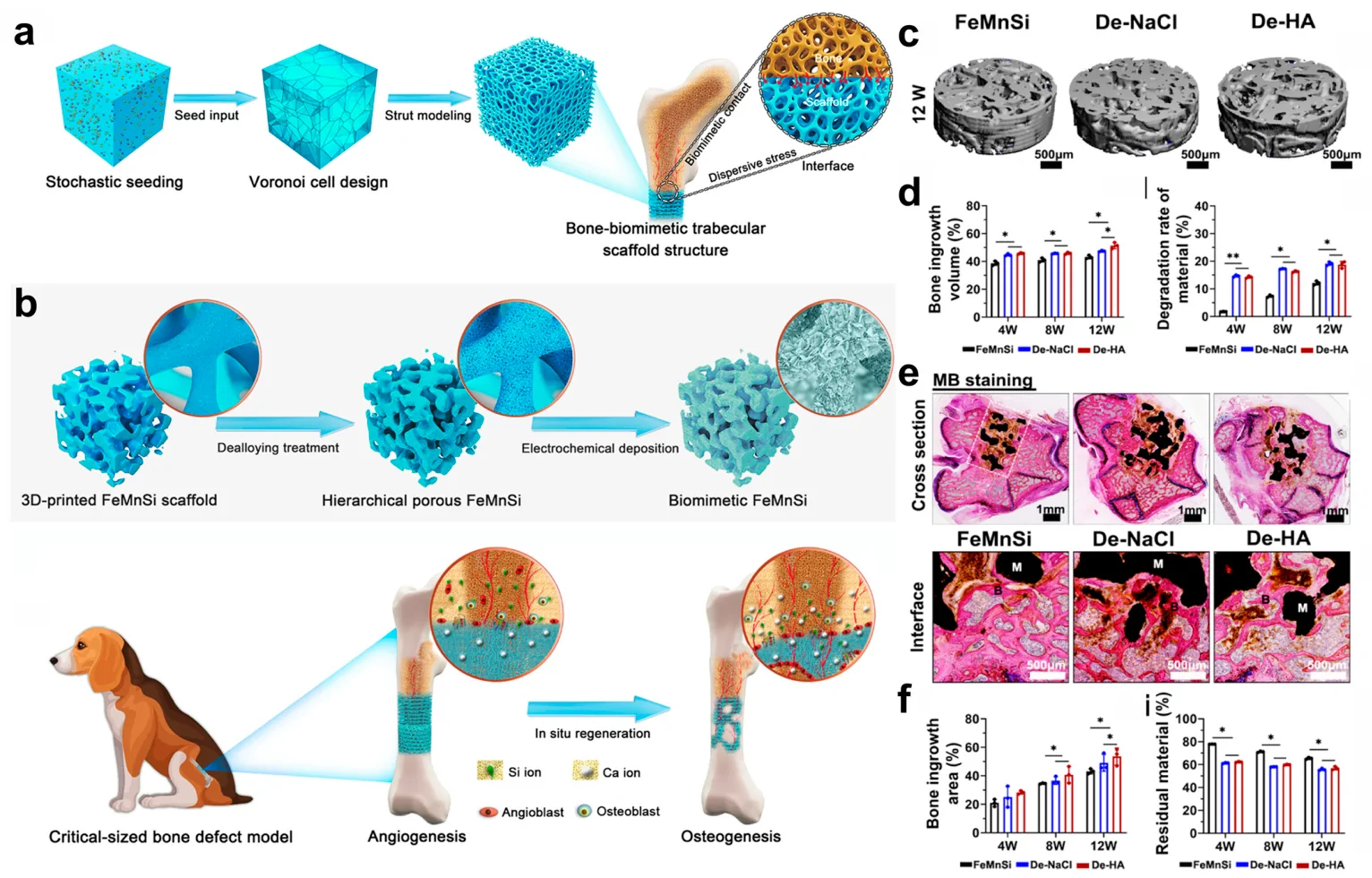
(**a**) Design of a stochastic Voronoi-based trabecular scaffold. (**b**) Fabrication of FeMnSi scaffold via 3D printing, dealloying, and electrochemical deposition, and its role in bone regeneration. (**c**) Micro-CT images of FeMnSi, De-NaCl, and De-HA scaffolds. (**d**) Analysis of bone ingrowth and degradation over time. (**e**) Histological (MB staining) evaluation of bone formation and interface integration. (**f**) Quantification of bone ingrowth area and residual material. * *p* < 0.05; ** *p* < 0.01. Reprinted with permission of the ref. [33].

Magnetic hydrogels have also been engineered at the microscale to mimic functional tissue units. However, routine handling of microtissues, such as during medium exchange, can compromise their structural integrity. To address this limitation, a recent article introduced on-chip magnetic microcryogels, a microscale hydrogel system incorporating MNPs [76]. These magnetic microcryogels enable precise, gentle manipulation of microtissues, reducing cell loss during culture, maintaining structural stability over extended periods, and enabling magnetic purification of specific functional units. In a recent study, a biomimetic, hierarchically micro–nanoporous FeMnSi scaffold was developed via a synergistic strategy combining 3D printing, dealloying, and electrochemical deposition to address critical-sized bone defect repair in load-bearing regions. As illustrated in Figure 4a, a stochastic Voronoi-based lattice design was employed to mimic trabecular bone architecture, enhancing stress transfer efficiency compared to conventional periodic structures. Figure 4b illustrates the fabrication workflow, in which a 3D-printed FeMnSi scaffold undergoes dealloying and electrochemical modification to achieve hierarchical porosity and biomimetic surface features, thereby promoting angiogenesis and osteogenesis in vivo. Microstructural comparisons in Figure 4c highlight the distinct porous morphology of the FeMnSi scaffold compared with the control groups (De-NaCl and De-HA). Quantitative analysis in Figure 4d shows improved bone ingrowth and a balanced degradation rate over time. Histological evaluation in Figure 4e (MB staining and interface imaging) confirms enhanced new bone formation and integration at the scaffold–bone interface. Finally, Figure 4f presents quantitative metrics of bone ingrowth area and residual material, demonstrating a dynamic equilibrium between scaffold degradation and tissue regeneration. Mechanistically, the scaffold promotes osteogenesis and angiogenesis by activating the EGFR-mediated Ras/Raf/MAPK signaling pathway, ultimately enabling vascularized bone regeneration and functional reconstruction in large animal models [33]. On the other hand, iron oxide nanoparticles also have potential for TE. A recent study presents a simple magnetic-field-assisted electrospinning method to fabricate highly aligned silk fibroin nanofibers by incorporating Fe_3_O_4_ nanoparticles into the spinning solution. The resulting scaffolds showed enhanced mechanical strength, magnetic responsiveness, and strong anisotropy, enabling them to guide MSC elongation, vascular-like differentiation, and oriented C_2_C_12_ myoblast growth. The work demonstrates that aligned silk nanofibers can be produced through a straightforward process, achieving performance comparable to that of more complex electrospinning techniques, highlighting their potential for skeletal muscle tissue engineering [28].

### 3.3. Magnetic Hyperthermia for Simultaneous Tumor Ablation and Bone Tissue Regeneration

Osteosarcoma and metastatic bone disease frequently necessitate surgical resection that creates large structural defects, presenting a dual clinical challenge that demands both effective oncological treatment and simultaneous regenerative reconstruction of load-bearing bone tissue [77,78]. In this context, magnetic hyperthermia, which exploits the localized heat generated by MNPs under an alternating magnetic field (AMF) to selectively damage tumor cells while preserving the integrity of surrounding healthy tissue, has emerged as a particularly compelling scaffold-integrated strategy, precisely because it can address both clinical demands within a single biomaterial platform [79].

A recently developed iron oxide–bioactive glass core–shell nanocomposite exemplifies this dual-function regenerative paradigm [80]. Designed from the outset as a tissue engineering scaffold rather than a standalone oncological agent, this architecture leverages two complementary material functions simultaneously: the iron oxide core provides magnetic heating capacity sufficient to destroy residual bone cancer cells under AMF exposure, whilst the bioactive glass shell promotes bonding to host bone tissue and stimulates mineralization, a key indicator of successful scaffold integration and the onset of functional bone regeneration [81]. Among tested formulations, higher calcium content in the bioactive glass component yielded the fastest mineralization rate and the strongest magnetic response, establishing a structure–property relationship with direct relevance to scaffold optimization for bone defect repair [82].

Within the tissue engineering workflow, the therapeutic temperature window of magnetic hyperthermia serves a sequential dual regenerative function. At ablative temperatures (>45 °C), irreversible apoptosis and necrosis are induced in tumor cells [83], clearing the defect site and creating a scaffold-ready environment for subsequent tissue ingrowth. At sub-ablative temperatures (41–43 °C), mild hyperthermia shifts from a destructive to a constructive role: rather than modulating cellular stress responses or potentiating chemotherapeutic efficacy alone [84], controlled thermal stimulation within MNP-laden scaffolds has been shown to actively stimulate osteogenic differentiation pathways, upregulate alkaline phosphatase activity, and enhance vascularization, all prerequisites for functional bone reconstruction [85]. Magnetic hyperthermia therefore operates sequentially within the regenerative workflow: ablative thermal dosing clears malignant tissue and prepares the defect site, whilst subsequent sub-ablative stimulation drives the osteogenic and angiogenic signaling required for structural bone regeneration.

The specific absorption rate (SAR), a measure of power dissipated per unit mass under a given AMF, governs thermal output and is determined by particle size, shape, crystallinity, and AMF parameters (frequency and amplitude) [86]. Optimizing the SAR within the geometric and porosity constraints of biocompatible scaffold architectures, without compromising the mechanical load-bearing performance essential in bone tissue engineering, remains the central engineering challenge in translating this dual-function approach to clinical practice [86].

It is important to note that magnetic hyperthermia is not limited to dissipative heating mechanisms arising from magnetization reversal under an alternating magnetic field. An alternative and physically distinct mechanism, ferromagnetic resonance, can also generate localized hyperthermia-range heating in magnetic nanoparticles [87]. In ferromagnetic resonance, energy absorption occurs at characteristic frequency-field product values of approximately 10^10^–10^11^ A/(m·s), substantially higher than the ~100 kHz and ~100 Oe conditions typically employed in conventional dissipative hyperthermia protocols, and overlapping with the frequency ranges used in magnetic resonance imaging [87]. This distinction carries practical relevance for tissue engineering scaffold design: while SAR-governed dissipative heating is determined primarily by particle size, shape, and crystallinity, ferromagnetic resonance-based heating introduces additional tunable parameters, including resonance frequency, applied field orientation, and anisotropy field that may offer complementary control over thermal dosimetry within MNP-laden scaffolds [88]. Integrating both mechanisms into the design framework for magnetic scaffolds, therefore, broadens the available parameter space for achieving the precise sub-ablative thermal stimulation required for osteogenic differentiation and vascularization, whilst remaining within the safety constraints imposed by the Brezovich limit for clinical field exposure [88].

## 4. Biocompatibility, Toxicology, and In Vivo Fate

### 4.1. Physicochemical Determinants of Biological Response and ROS-Mediated Cytotoxicity

Integrating MNPs into skeletal muscle tissue engineering poses significant translational hurdles, particularly regarding biocompatibility and the mitigation of long-term cytotoxicity [89,90]. The biological fate and cellular response to MNPs are inextricably linked to their physicochemical properties; variables such as primary particle size, core composition, surface charge, and aggregation propensity within physiological media heavily dictate their interaction with biological systems. Extensive literature demonstrates that bare, uncoated, or inadequately functionalized MNPs can provoke severe intracellular damage. Specifically, the degradation of iron-based MNP cores can catalyze Fenton-like reactions, triggering the excessive generation of reactive oxygen species (ROS), subsequent oxidative stress, and ultimately, apoptotic cell death [91,92]. This mechanism is particularly consequential in skeletal muscle, where the high metabolic rate and mitochondrial density create a baseline oxidative environment that amplifies nanoparticle-induced ROS burden.

Beyond direct oxidative damage, the subcellular localization of internalized MNPs critically disrupts essential cellular machinery. Nanoparticles that traffic to perinuclear compartments have been shown to aggregate proximal to or within mitochondria, impairing electron transport chain function, dissipating membrane potential, and inducing mitophagy and generalized autophagy [93]. In highly energy-demanding skeletal muscle fibers, which are critically dependent on mitochondrial ATP production for both contractile function and regenerative capacity, such mitochondrial perturbation carries an elevated risk of chronic metabolic dysfunction and impaired myogenic recovery.

### 4.2. Surface Engineering Strategies for Toxicity Mitigation

To circumvent these adverse bio-interactions, robust surface functionalization is imperative. Encapsulating the magnetic core with biocompatible hydrophilic shells, including polyethylene glycol (PEG), dextran, and zwitterionic polymers, or integrating MNPs into advanced nanocomposites, markedly improves colloidal stability and shields reactive core surfaces from non-specific protein adsorption and receptor-mediated cytotoxicity [94]. The physicochemical rationale is well established: hydrophilic coronas reduce interfacial energy, suppress aggregation in ionic physiological media, and sterically occlude the iron oxide surface from direct contact with cellular lipid bilayers and soluble iron-responsive proteins. Recent advances have shifted toward sophisticated hybrid paradigms that extend beyond simple shell coatings. These include biodegradable polymeric matrices (e.g., PLGA, polycaprolactone), structural hydrogel scaffolds that spatially confine MNPs within an engineered extracellular matrix analog, and biomimetic cell-membrane coatings derived from erythrocytes or macrophage membranes, which confer superior immune evasion through self-recognition signaling [95]. Cell-membrane camouflage strategies are particularly promising for skeletal muscle applications, as they substantially reduce macrophage-mediated phagocytic clearance at the implantation site, prolonging therapeutic residence while minimizing inflammatory activation.

**Figure 5:**
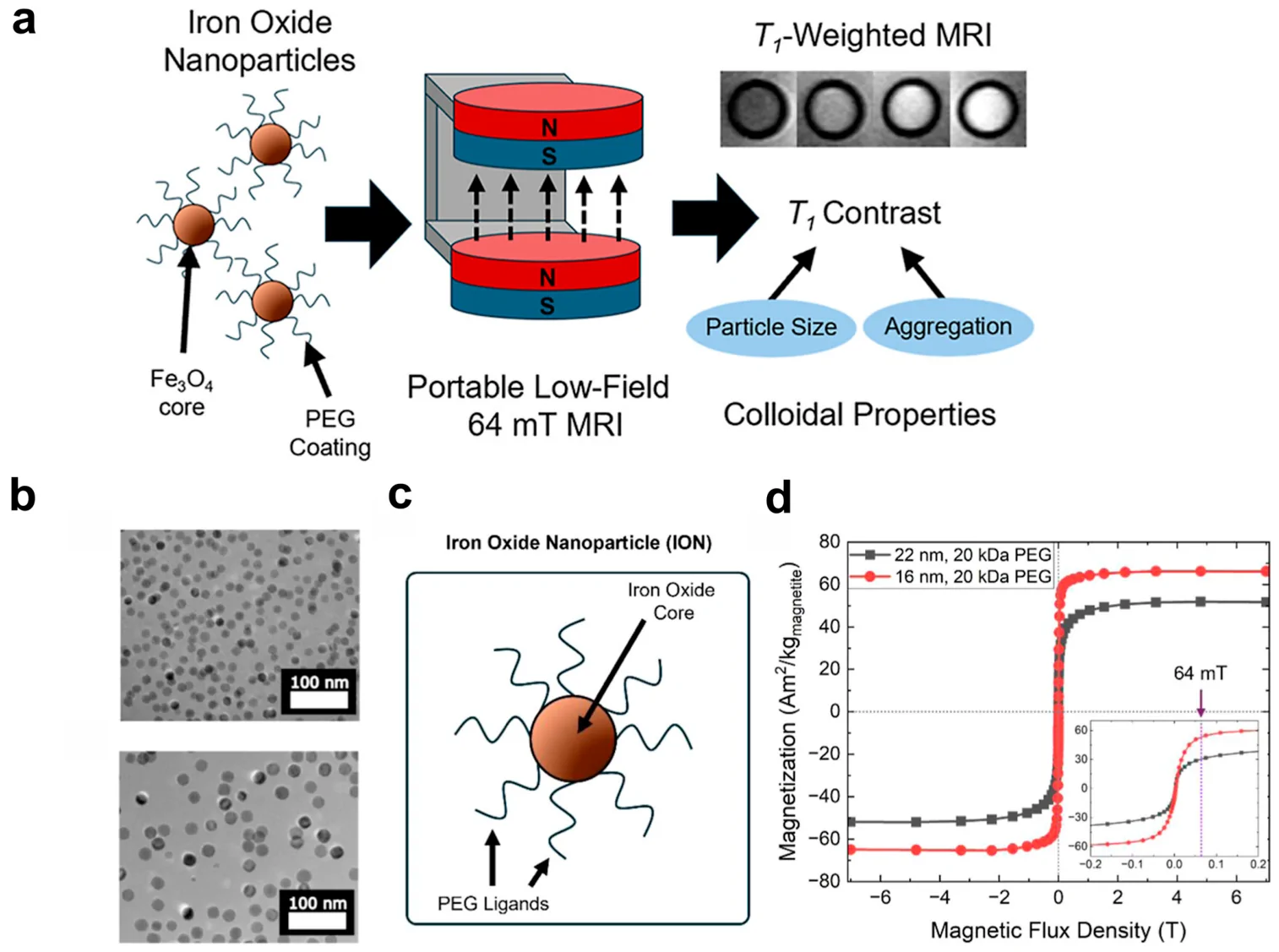
(**a**) Schematic of PEG-coated Fe_3_O_4_ nanoparticles designed for enhanced T_1_ contrast at 64 mT. (**b**) TEM images of monodisperse 16 and 22 nm IONPs. (**c**) Magnetization curves showing the low-field magnetic response of both particle sizes. (**d**) Low-field MRI workflow illustrating how nanoparticle size, colloidal stability, and aggregation influence T_1_-weighted contrast at 64 mT. Reprinted with permission of the ref. [96].

For low-field MRI applications, surface engineering serves a dual purpose, reducing toxicity whilst simultaneously optimizing contrast performance. Oberdick et al. demonstrated that monodisperse Fe_3_O_4_–PEG nanoparticles (16 and 22 nm core diameters) engineered for operation at 64 mT exhibit longitudinal relaxivity exceeding clinical gadolinium-based agents by over an order of magnitude (Figure 5) [96]. Critically, this study established that colloidal stability is a primary determinant of T_1_ performance: particles functionalized with shorter PEG chains displayed greater aggregation, directly reducing r_1_ values. NMRD profiling confirmed that Néel relaxation dominates the low-field relaxation mechanism, with particle size and magnetic anisotropy jointly governing relaxivity. These findings underscore that the same surface engineering principles required for biocompatibility, minimizing aggregation, and maintaining monodispersity are simultaneously necessary for maximizing diagnostic utility in portable 64 mT MRI systems.

### 4.3. In Vivo Biodistribution, RES Sequestration, and Organ-Specific Accumulation

The systemic administration of MNPs introduces critical translational challenges regarding in vivo biodistribution and clearance kinetics [97]. Following intravenous exposure, MNPs are rapidly opsonized by serum proteins, predominantly immunoglobulins, complement components, and apolipoproteins, forming a protein corona that fundamentally reprogrammes their cellular uptake profile and targeting efficiency [98]. This corona formation is rapid, occurring within seconds to minutes of blood contact, and confers a dominant “eat-me” signal recognized by pattern-recognition receptors on tissue-resident and circulating phagocytes of the mononuclear phagocyte system, more commonly referred to as the reticuloendothelial system (RES).

Organ-level accumulation is consequently highly non-uniform and governed by both vascular architecture and local macrophage density. Because renal filtration is generally restricted to particles below approximately 8 nm or to specifically engineered renal-clearable constructs, the majority of clinically relevant MNP formulations bypass glomerular filtration and accumulate heavily in tissue-resident Kupffer cells of the liver and marginal zone macrophages of the spleen [99]. Quantitative biodistribution studies consistently report hepatic and splenic MNP burdens that dwarf those in other organs during the acute and subacute phases following systemic exposure. This RES tropism, while partially protective in confining nanoparticle distribution, simultaneously concentrates iron loading in organs with limited capacity for high-throughput iron metabolism beyond physiological norms.

Prolonged retention in these filtration organs raises substantive concerns of localized iron overload, dysregulated ferritin and transferrin saturation, secondary ROS generation via Fenton chemistry within lysosomal compartments, and long-term cellular toxicity in hepatocytes and splenic stromal cells [100]. Off-target accumulation in additional organs, including the lungs, bone marrow, and lymph nodes, further complicates the systemic toxicity profile, particularly under conditions of repeated or high-dose administration.

### 4.4. Clearance Kinetics and the Biodegradable Design Paradox

To mitigate chronic RES overload and organ toxicity, researchers are actively engineering MNPs with bioresorbable polymer coatings that fulfill a dual function: shielding the reactive iron oxide core to limit premature protein adsorption and inflammatory activation during circulation, whilst facilitating controlled endolysosomal degradation following cellular internalization, a prerequisite for safe long-term drug delivery and therapeutic applications [101].

The degradation sequence is mechanistically well defined. Once internalized into acidic endolysosomal compartments (pH 4.5–5.5), the protective polymer shell undergoes hydrolytic or enzymatic degradation at a rate determined by the specific coating chemistry polyester linkages (PLGA, PLA) degrade via bulk hydrolysis on a timescale of weeks to months, whilst dextran coatings are susceptible to lysosomal dextranase activity on shorter timescales [102]. Progressive shell dissolution exposes the iron oxide core to proton-mediated dissolution and native hydrolytic enzymes, releasing soluble Fe^2+^/Fe^3+^ ions that enter the endogenous iron recycling pool (ferritin storage → transferrin transport → hemoglobin synthesis). When the rate of iron liberation remains within physiological buffering capacity, this pathway supports complete metabolic clearance without systemic iron accumulation.

This degradation kinetics, however, highlights the fundamental engineering paradox that currently constrains the field: structural and magnetic stability, essential for an adequate therapeutic window, targeting efficiency, and diagnostic signal, are mechanistically in tension with rapid, predictable biodegradability. Coatings sufficiently robust to maintain colloidal integrity and resist premature corona-driven opsonization during circulation inevitably slow lysosomal degradation, thereby extending organ retention. Conversely, rapidly degradable formulations that clear efficiently tend to exhibit inferior magnetic performance and reduced circulation half-life. Resolving this paradox requires coating architectures that are conditionally stable, resistant to the neutral pH and protease-poor environment of the bloodstream, but selectively labile within the acidic, enzyme-rich lysosomal milieu, an active frontier in stimuli-responsive nanoparticle design.

### 4.5. Critical Gaps: The Need for Long-Term Longitudinal In Vivo Evaluation

Despite the material innovations described above, the field continues to suffer from a conspicuous deficit of comprehensive, long-term in vivo evaluations. The preponderance of published biocompatibility assessments remains limited to acute (24–72 h) or subchronic (up to 30 days) cytotoxicity endpoints in rodent models, which are structurally inadequate to capture chronic biodistribution trajectories, cumulative organ burden, and delayed toxicological sequelae most relevant to clinical translation.

This gap is especially consequential for skeletal muscle tissue engineering applications, where MNP-augmented constructs are intended to persist within regenerating muscle beds for weeks to months, and where off-target MNP migration to metabolically active vital organs, such as the liver, spleen, kidney, and cardiac muscle, could impose compounding iron loading over extended timeframes. Extensive longitudinal studies spanning months to years in appropriate large-animal models remain an absolute prerequisite for accurately decoding the chronic biodistribution, clearance kinetics, and systemic toxicity of MNP-augmented grafts. Such studies must simultaneously evaluate: (i) temporal changes in organ-specific iron accumulation and speciation; (ii) the kinetics of polymer shell degradation in situ; (iii) inflammatory and fibrotic responses in both the target tissue and RES organs; and (iv) functional outcomes in skeletal muscle regeneration and contractile performance. Until this longitudinal evidence base is established, the translational pathway for MNP-integrated skeletal muscle constructs remains incompletely defined.

## 5. Limitations in Extensive Clinical Applications

The clinical translation of MNPs for skeletal muscle tissue engineering faces interconnected biological, technological, and regulatory barriers that must be collectively addressed. A foundational challenge is the absence of standardized protocols for MNP synthesis, characterization, and functionalization, whereby inconsistent surface coatings, magnetic behavior, and batch-to-batch variability produce unpredictable therapeutic performance and safety profiles [103,104]. Regulatory agencies, including the Food and Drug Administration (FDA) and European Medicines Agency (EMA), consequently demand rigorous quality-control frameworks and pharmaceutical-grade reproducibility as prerequisites for clinical authorization, standards that the field has not yet uniformly achieved [104]. On the manufacturing side, scalable platforms such as continuous-flow microreactor synthesis and automated quality-control systems are being progressively adopted to improve batch consistency and support cGMP compliance, though their implementation remains uneven [105,106].

Biological barriers present equally significant translational constraints. Species-dependent differences in cellular uptake, immune activation, and nanoparticle processing reduce the predictive validity of preclinical animal models for human outcomes [107,108,109,110]. While the long-term biocompatibility, chronic biodistribution, and potential toxicity of MNPs in human tissues remain insufficiently characterized [111,112]. Most existing safety datasets derive from acute or subchronic rodent studies that are structurally inadequate to detect delayed adverse events, including hepatic iron overload, splenic fibrosis, or latent genotoxicity relevant to sustained clinical use. Ethical constraints under increasingly stringent 3Rs regulations further complicate this landscape by limiting access to the longitudinal large-animal studies that would generate the most clinically informative safety evidence. The complexity of MNP synthesis and the continued absence of universally adopted reference standards further hinder inter-laboratory reproducibility and undermine the cross-study evidence-based regulatory submissions required.

Despite these challenges, the field is making substantive progress. Biodegradable and surface-engineered nanoparticle formulations are improving physiological clearance and reducing long-term tissue accumulation, while advances in computational modeling and real-time magnetic field feedback systems are refining stimulation parameters to optimize muscle regeneration outcomes. Realizing the full clinical potential of MNP-based strategies will ultimately require sustained and formally structured collaboration among materials scientists, bioengineers, clinicians, and regulatory bodies to establish unified translational frameworks from standardized preclinical data packages through to validated first-in-human trial designs that align scientific innovation with regulatory expectations from the earliest stages of MNP development.

## 6. Conclusions and Future Directions

Surface-engineered magnetic nanoparticles have emerged as a genuinely multifunctional platform for skeletal muscle tissue engineering, integrating controlled cell organization, magnetically guided therapeutic delivery, scaffold-level mechano-biological stimulation, and non-invasive MRI-based construct monitoring within a single material system. This review has traced that trajectory across four interconnected domains: surface engineering strategies governing colloidal stability, immune evasion, and magnetic performance; magnetically responsive platforms for myogenic cell labeling, non-invasive monitoring, and controlled cargo delivery; scaffold-integrated magnetic systems for skeletal muscle, bone, cardiac, and neural tissue regeneration; and the biocompatibility and biodistribution challenges that continue to define the gap between preclinical promise and clinical implementation.

Several technical priorities emerge clearly from this synthesis. The engineering paradox identified in Section 4.4, wherein coating stability sufficient for adequate circulation half-life and therapeutic performance is mechanistically in tension with the rapid lysosomal degradation required for safe clearance, remains the central unsolved problem in MNP design for skeletal muscle applications. Conditionally stable coatings that are resistant at physiological pH but selectively labile within acidic endolysosomal compartments represent the most promising design direction for resolving this tension. For skeletal muscle specifically, where the high metabolic rate and mitochondrial density create a baseline oxidative environment that amplifies nanoparticle-induced ROS burden, surface-engineering decisions carry greater consequences: inadequately functionalized MNPs risk mitochondrial perturbation, impaired electron transport chain function, and chronic disruption of myogenic recovery capacity.

The field’s most consequential structural gap remains the near absence of long-term, longitudinal in vivo safety data. Acute and subchronic rodent studies dominate the existing biocompatibility literature and are structurally insufficient to characterize chronic biodistribution trajectories, cumulative iron loading in RES organs, and delayed inflammatory or fibrotic responses, all of which are most relevant to skeletal muscle constructs intended to persist within regenerating tissue beds for weeks to months. Large-animal longitudinal studies spanning months to years, simultaneously evaluating organ-specific iron accumulation, polymer shell degradation kinetics, inflammatory responses in both target muscle and RES organs, and functional contractile performance outcomes, are absolute prerequisites for a credible translational pathway.

Manufacturing and regulatory barriers compound these biological challenges. Batch-to-batch variability, the absence of universally adopted reference standards, and inconsistent characterization practices collectively undermine the cross-study reproducibility demanded by the FDA and EMA for clinical authorization. The progressive adoption of continuous-flow microreactor synthesis and automated quality-control systems is encouraging, but implementation remains uneven.

Looking forward, the convergence of surface-engineered MNP platforms with aligned electrospun scaffolds, injectable magnetic hydrogels for direct muscle delivery, wearable and implantable magnetic field devices, and real-time closed-loop feedback systems offers a compelling vision for adaptive, personalized skeletal muscle regeneration therapies. The integration of artificial intelligence for predictive modeling of nanoparticle behavior in complex physiological environments may further accelerate the rational design of next-generation MNP-augmented muscle constructs. Realizing these prospects will require formally structured collaboration among materials scientists, bioengineers, clinicians, and regulatory bodies, beginning at the earliest stages of MNP development rather than at the point of clinical submission.

## Figures and Tables

**Table 1:** Surface-engineered MNPs for tissue engineering and regenerative medicine applications.

Category	Agent	Platform	Conjugation	Target	Model	Outcome	Ref.
Scaffold	Fe_3_O_4_ nanoparticles	Silk fibroin/Fe_3_O_4_ electrospun nanofibers	Magnetic field-assisted electrospinning	Skeletal muscle/MSCs	In vitro C_2_C_12_ myoblasts	Guided cell alignment, myogenic differentiation, enhanced mechanical strength	[28]
Scaffold	Ferrofluid	Ferrofluid-based bioink for 3D bioprinting	Embedded in bioink matrix	Skeletal muscle tissue	In vitro muscle constructs	Enhanced force generation and magnetic responsiveness	[29]
Cell Delivery	Fe_3_O_4_ nanoparticles	Magneto-responsive semi-IPN hydrogel microbeads	Microfluidic pressure-driven gelation	Cell delivery platform	In vitro cell adhesion and proliferation	Magnetically guided cell delivery, tunable swelling, superior mechanical elasticity	[30]
Therapeutic Payload	Curcumin + Fe_3_O_4_	N-isopropylacrylamide–methacrylic acid hydrogel	Encapsulation in thermoresponsive hydrogel	Cardiac muscle	In vitro cardiomyocyte cell lines	Reduced doxorubicin-induced cardiotoxicity	[31]
Scaffold	Fe_3_O_4_ nanoparticles	Ferrogel–alginate scaffold	Periodic magnetic stimulation (5 min, 1 Hz, every 12 h)	Skeletal muscle	In vivo rodent model	Mechanically activated biomaterial; enhanced muscle repair	[32]
Bone Regeneration	FeMnSi alloy	3D-printed Voronoi-lattice FeMnSi scaffold	Dealloying + electrochemical deposition	Bone defect	Large animal model	Vascularized bone regeneration via EGFR/Ras/Raf/MAPK pathway	[33]

**Table 2:** Surface-engineered MNPs for theranostic and imaging applications.

Category	Agent	Platform	Conjugation	Target	Model	Outcome	Ref.
Targeting ligand	PSMA ligand	PEG-modified ferrite NPs	Flexible linked to PSMA	PSMA	PSMA tumor model	Enhance delivery of MNPs	[56]
Targeting ligand	Hyaluronic acid (HA)	Fe_3_O_4_ core; HA-FeWO_4_ NPs	HA surface functionalization	CD44 receptor	4T1 breast cancer cells	PTT + MRI/CT multimodal imaging	[57]
Therapeutic Payload	MRI + Fe_3_O_4_ core	High-magnetic-moment iron oxide nanoparticles	PEG + drug surface loading	Tumor microenvironment	In vivo cancer theragnostic model	MRI-guided magneto-chemotherapy	[49]
Therapeutic Payload	Doxorubicin (DOX)	DOX-TFP-MNPs	Encapsulation: TFP polymer shell on magnetic core	PC3 prostate xenograft	NOD SCID mice bearing PC3-KD xenografts	pH/thermo-triggered DOX release	[58]
Therapeutic Payload	Curcumin (CUR)	SPIONs modified with alendronate-β-cyclodextrin	Host–guest crosslinking via β-cyclodextrin on SPION surface	Breast cancer	In vitro + in vivo tumor models	Tumor size reduction; superior T_2_ MRI contrast performance	[59]
Therapeutic Payload + Targeting	Curcumin (CUR) + Gadolinium ions (Gd^3+^)	Fe_3_O_4_ NPs coated with polycyclodextrin functionalized with Gd^3+^	Polycyclodextrin coating on Fe_3_O_4_ + Gd^3+^ chelation + CUR encapsulation	4T1 cells	MCF10A; BALB/c mice bearing 4T1 xenografts	Combined T_1_/T_2_ dual-mode MRI contrast + curcumin chemotherapy	[60]
Imaging agent	Macrophage membrane	Porous Fe_3_O_4_ magnetic NPs encapsulated in macrophage membrane	Biomimetic cell membrane camouflage coating on porous magnetic core	Glioma tumor cells (C6 glioma)	Rats bearing C6 glioma xenografts	Tumor homing; multimodal MRI + fluorescence imaging;	[61]
Imaging agent	PEG coating + Au shell (Janus structure)	Fe_3_O_4_–Au shell Janus nanostructures (Fe_3_O_4_–Au shell-PEG)	PEG surface modification + Au shell engineering on Fe_3_O_4_ core	Breast cancer (MRI contrast + PTT)	Breast cancer model	Synergistic MRI + PTT	[62]

## Data Availability

No new data were created or analyzed in this study. Data sharing is not applicable to this article.
